# Detection and complete genome characterisation of bat coronaviruses from Ghana

**DOI:** 10.1007/s00705-026-06628-y

**Published:** 2026-05-21

**Authors:** Philip El-Duah, Richmond Yeboah, Julia Melchert, William Tasiame, Emmanuella Nyarko-Afriyie, Augustina Angelina Sylverken, Michael Owusu, Yaw Adu-Sarkodie, Christian Drosten, Victor Max Corman

**Affiliations:** 1 Institute of Virology, Charité-Universitätsmedizin Berlin, Free University, Humboldt-University, Berlin Institute of Health, Berlin, Germany; 2 Kumasi Centre for Collaborative Research in Tropical Medicine, Kwame Nkrumah University of Science and Technology, Kumasi, Ghana; 3 School of Veterinary Medicine, Kwame Nkrumah University of Science and Technology, Kumasi, Ghana; 4 Department of Theoretical and Applied Biology, Kwame Nkrumah University of Science and Technology, Kumasi, Ghana; 5 Department of Medical Diagnostics, Kwame Nkrumah University of Science and Technology, Kumasi, Ghana; 6 Department of Clinical Microbiology, Kwame Nkrumah University of Science and Technology, Kumasi, Ghana

## Abstract

Alphacoronaviruses comprise human, livestock, and wildlife coronaviruses of importance, particularly to the livestock industry worldwide. Domestic livestock have the potential to serve as intermediate hosts of coronaviruses, given the contact they have with both humans and wildlife. With the aim of detection and subsequent complete genome sequencing of novel and known but previously undetected alphacoronaviruses, a screening RT-PCR assay was established. In vitro transcripts of two human alphacoronaviruses were generated to evaluate assay performance, and a collection of archived bat and livestock viral RNA samples was tested as part of assay validation. Confirmation of RT-PCR-positive outcomes was done by Sanger and high-throughput sequencing, before phylogenetic analysis. The assay can detect both human alphacoronaviruses with a detection limit of 46 copies/reaction of *Alphacoronavirus amsterdamense* and 504 copies/reaction of *Alphacoronavirus chicagoense *in vitro transcripts, as estimated by a probit regression analysis. Validation screening of domestic and peri-domestic livestock species, including cattle, sheep, goats, donkeys, pigs, rabbits, and grasscutters, did not yield any positive outcomes. Confirmed detections were made in bats (3.11%), comprising 1.84% alphacoronaviruses and 1.27% betacoronaviruses. Two complete genomes were obtained for an *Alphacoronavirus chicagoense*-related bat coronavirus and a putative member of the subgenus *Hibecovirus*. A near-complete genome was obtained for a *Chaerephon* spp. bat alphacoronavirus, which was found to be most closely related to a similar virus from Nigeria. This study presents a sensitive assay for coronavirus surveillance and provides additional complete genomes, thereby enhancing our understanding of coronavirus diversity.

**Supplementary Information:**

The online version contains supplementary material available at 10.1007/s00705-026-06628-y.

## Introduction

Coronaviruses are enveloped viruses with positive-strand RNA genomes of size ranging from 24 to 32 kilobases and are pathogenic to a range of hosts, including mammals and birds. Coronaviruses belong to the family *Coronaviridae* in the order Nidovirales and are classified into four genera, namely *Alphacoronavirus*, *Betacoronavirus*, *Gammacoronavirus*, and *Deltacoronavirus* [1]. The genus *Betacoronavirus* is known for important members, including *Betacoronavirus pandemicum* (both Severe Acute Respiratory Syndrome Coronaviruses or SARS-CoVs) and *Betacoronavirus cameli* (Middle East Respiratory Syndrome Coronavirus or MERS-CoV), which have been responsible for major epidemics and most recently a pandemic [2]. Despite the focus on betacoronaviruses due to their role in recent emergence events, alphacoronaviruses also include important viruses of humans, as well as wild and domesticated animals that need to be studied. Human alphacoronaviruses include the common cold viruses *Alphacoronavirus chicagoense* (Human coronavirus 229E or HCoV-229E) and *Alphacoronavirus amsterdamense* (Human coronavirus NL63 or HCoV-NL63), which are endemic in human populations worldwide [3, 4]. Animal alphacoronaviruses include those of domestic pets, such as canine coronavirus of dogs and feline coronavirus of cats. Among livestock, pigs are the most important hosts for alphacoronaviruses, and these include *Alphacoronavirus porci* (Porcine epidemic diarrhea virus or PEDV), *Alphacoronavirus suis* (Transmissible gastroenteritis virus or TGEV), porcine respiratory coronavirus (PRCV), and a more recently discovered *Alphacoronavirus rhinolophi* – related Swine acute diarrhea syndrome coronavirus (SADS-CoV) [5–7]. The alphacoronaviruses in pigs, especially *A. porci*, are of economic importance due to the devastation they have caused in the swine rearing industry. In Asia and the United States, substantial economic losses have been incurred due to outbreaks of *A. porci* in livestock farms [8, 9]. Wild animals such as bats and rodents have been found to harbor diverse coronaviruses worldwide [10–12]. Bats have been identified as the source of most human coronaviruses that also infect intermediate livestock hosts, as in the case of *B. cameli* and dromedary camels [13, 14] and the first *B. pandemicum* (SARS-CoV), which infected Himalayan civet cats [15, 16]. Surveillance studies focused on intermediate hosts will provide further insights into the host range and biogeography of coronaviruses with zoonotic potential. Furthermore, detection of novel alphacoronaviruses in species other than bats provides insights into coronavirus emergence, such as the case of *A. chicagoense*-related bat coronaviruses in camelids [17]. The lack of any known intermediate host of *A. amsterdamense* [18] is also a gap in our knowledge of the natural history of coronaviruses. Coronaviruses, like many RNA viruses, undergo rapid evolution, mainly driven by mutations introduced during replication [19]. This rapid evolution also presents a challenge for molecular diagnostics. With time and continuous change, escape variants accumulate, and screening and detection assays must be updated accordingly. This challenge highlights the necessity for new, purposive assays to study coronaviruses of interest, which is one of the aims of this study, in which we focus on alphacoronaviruses that have been previously detected in Ghanaian bats [12]. Current nucleic acid-based methods capable of detecting alphacoronaviruses span a spectrum from broad, pan-coronavirus reverse transcription polymerase chain reaction (RT-PCR) assays that by nature of design, can detect alphacoronaviruses in both human and animal samples [20, 21], to more targeted assays specific for individual alphacoronaviruses either singly or multiplexed [22, 23]. Additionally, field-deployable techniques such as loop-mediated isothermal amplification (RT-LAMP), which are particularly suited for veterinary applications [24, 25], are also available. Broad, family-level assays typically tend to sacrifice some sensitivity compared to virus- or genus-specific assays; however, they remain valuable tools for large-scale screening and the discovery of both known and novel coronaviruses. Continuous surveillance of coronaviruses at the whole genome level is also essential for understanding the evolution of this important virus family. A lot of unclassified coronaviruses persist, particularly from Africa, due to a lack of complete genome sequences, which hinders further taxonomic classification [26]. This study sought to contribute a new RT-PCR detection approach for alphacoronaviruses suitable for multi-species surveillance, and to provide additional data at the complete genome level on some circulating coronaviruses in Ghana. By this, we aim to provide high-resolution insights into the genetic diversity of coronaviruses in Ghana and, more broadly, their global evolutionary relationships, thereby supporting improved surveillance strategies.

## Materials and methods

### PCR primer design

**Fig. 1:**
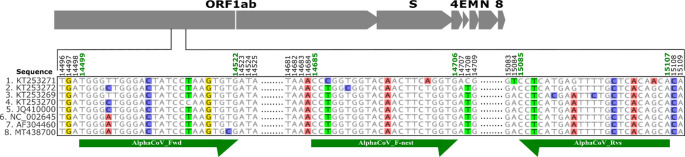
Schematic representation of primer binding sites. A representative coronavirus genome, including open reading frames 1ab (ORF1ab), 4, and 8, as well as the spike (S), envelope (E), membrane (M), and nucleocapsid (N) regions, is shown with the primer-binding sites indicated. Forward and reverse primer positions are mapped to conserved regions identified during primer design. The first eight sequences in the alignment are labeled by their accession numbers. Sequence ambiguities are highlighted using colored nucleotide bases. Primer start and end positions are indicated in green text, corresponding to green arrows, while arrowheads denote the 3′ orientation of each primer

A total of 1122 complete and partial alphacoronavirus genomes were downloaded from NCBI in August 2022. Unverified sequences and cDNA clones were excluded from the search result, and duplicates were also removed. The RdRP sequences were selected, and the rest of the genome was deleted. The RdRP was targeted due to the high level of conservation across coronaviruses in this region. The extracted sequences were aligned in Geneious Prime 2020 (https://www.geneious.com*)*, by the Multiple Alignment using Fast Fourier Transform (MAFFT) algorithm (Online Resource 1). Forward and reverse primer pairs were then selected by manual inspection of the genome for conserved regions. Positions in the primers corresponding to regions of high polymorphism in the genome were represented by degenerate bases according to the International Union of Pure and Applied Chemistry (IUPAC). Calculation of primer melting temperatures was done by the nearest neighbor method [27]. Selection of a nested forward primer was based on an alignment of 116 *A. amsterdamense* - and *A. chicagoense* -related sequences from bats, alpacas, humans, and camels (Online Resource 2; Fig. 1) to increase the likelihood of detecting these groups of viruses with minimal primer mismatches. In silico analysis based on an alignment of 69 betacoronaviruses (Online Resource 3) was performed to evaluate potential cross-genus binding of primers beyond alphacoronaviruses.

### Generation of in vitro transcripts

Standardized and quantified controls were generated by in vitro transcription for *A. chicagoense* and *A. amsterdamense* in the region spanning positions 14,469 to 15,159 of the reference genomes. Synthetic double-stranded DNA fragments with a terminal T7 promoter and including the fragment region of interest were designed based on reference sequences from NCBI (accession numbers: NC_002645.1 and NC_005831.2) and synthesised (Integrated DNA technologies, Leuven, Belgium). In vitro transcription was done using the MEGAscript T7 Kit (Thermo Fisher Scientific, MA, USA) according to manufacturer’s instructions. The in vitro transcription product was purified by a phenol-chloroform extraction approach [28] using TRIzol™ LS Reagent (Thermo Fisher Scientific, MA, USA). Briefly, in vitro transcription product was mixed with TRIzol™ reagent at a 1:3 ratio. The mixture was incubated for 5 minutes, then mixed with chloroform. After centrifugation, the aqueous supernatant was removed and precipitated with isopropanol. RNA pellets were then resuspended in RNase-free water. The amount of RNA present after clean-up was determined using the Qubit™ RNA High Sensitivity kit (Thermo Fisher Scientific, MA, USA) according to manufacturer’s instructions, and the copy number concentration was calculated using the length of the RNA fragments and the measured amount [29].

### Establishment of hemi-nested RT-PCR

Selected primers were tested on in vitro transcripts (IVTs) in serial dilutions ranging from 5 × 10^6^ copies/reaction to 5 × 10^0^ copies/reaction in a one-step reverse transcriptase PCR assay to assess the limit of detection of primer combinations. Based on this, three primers were selected; two terminal primers and one nested primer that detected the lowest number of RNA copies. Different cycling conditions were tested, and the most conducive conditions were selected. Primers selected for the hemi-nested PCR were AlphaCoV_Fwd: TGGGHTGGGAYTAYCCHAARTGYG, AlphaCoV_Rvs: TGYTGNGARCARAAYTCATGWGG and AlphaCoV_F-nest: CCWGGTGGTACRACTTCTGGTG. These primers would yield a final amplicon of 419 base pairs (bp) in the second round. The first-round primer positions were located in a similar region as previously reported [30] and would promote pan-coronavirus detection. To estimate the limit of detection of the assay, serial 10-fold dilutions of the developed IVTs were made and tested in replicate. A probit regression analysis to determine the limit of detection with 95% probability of target detection was done using the R statistical package version 4.2.1.

RT-PCR reactions were performed in 25 µl volumes. First round mastermix was prepared by combining 3.1 µl of RNase-free water, 1 µl of bovine serum albumin (1 mg/ml), 0.4 µl MgSO_4_ (50 mM), 12.5 µl of Invitrogen 2X reaction mix, a buffer containing 0.4 mM of each deoxyribonucleotide triphosphates (dNTP) and 3.2 mM MgSO_4_ (Thermo Fisher Scientific, MA, USA), 0.5 µl each of the forward and reverse primers (10 µM) and 0.5 µl of SuperScript III One-Step RT-PCR enzyme with Platinum Taq polymerase (Thermo Fisher Scientific, MA, USA). A volume of 5 µl of template RNA was used.

The first-round amplification was performed by a reverse transcription step at 50 °C for 20 min, then an initial denaturation at 95 °C for 3 min. This was followed by 20 cycles of denaturation at 95 °C for 20 s, a touchdown annealing step starting from 60 °C for 20 s, then reducing by 0.5 °C per cycle and an extension step at 72 °C for 30 s. This was followed by another 30 cycles of denaturation at 95 °C for 20 s, annealing at 50 °C, extension at 72 °C for 30 s, and a final extension also at 72 °C for 2 min.

Mastermix for the second round PCR was prepared by combining 17.9 µl of RNase-free water, 0.2 µl MgCl_2_ (50 mM), 6.25 µl of 10X PCR buffer, containing 200 mM Tris HCl (pH 8.4) and 500 mM KCl (Thermo Fisher Scientific, MA, USA), 0.5 µl of dNTPs (10 mM each), 1 µl each of the forward and reverse primers (10 µM) and 0.1 µl of Taq polymerase enzyme (Thermo Fisher, MA, USA). A volume of 1 µl of template DNA generated from the first reaction was used. The second-round amplification conditions were the same as for the first round, without the reverse transcription step.

### Validation of established RT-PCR assay

The assay was evaluated by testing archived RNA extracts, stored at -80 °C and consisting of 388 rectal swabs from livestock collected from June 2015 to May 2016, comprising cattle (*n* = 147), sheep (*n* = 35), goats (*n* = 49), donkeys (*n* = 14), and pigs (*n* = 91) as well as faecal samples from farmed grasscutters (*n* = 30) and livers from rabbits (*n* = 22), collected between September 2019 and April 2021. Viral RNA from Bat faecal samples (*n* = 706) from 24 different species collected from February 2008 to June 2009 in Ghana were also screened with the assay. An overview of the samples included in assay validation is shown in Table 1.

**Table 1:** Overview of archive samples used in the study

Species	Number (%)	Sample type	Collection site (Region)	Total
Cattle	49	Rectal swab	Greater Accra	147
	42		Upper East	
	56		Volta	
Sheep	21	Rectal swab	Upper East	35
	14		Volta	
Goats	28	Rectal swab	Upper East	49
	21		Volta	
Donkeys	14	Rectal swab	Upper East	14
Pigs	42	Rectal swab	Ashanti	91
	21		Greater Accra	
	21		Upper East	
	7		Volta	
Grasscutters	24	Faeces	Ashanti	30
	6		Bono	
Rabbits	14	Liver	Ashanti	22
	5		Greater Accra	
	2		Eastern	
	1		Western	
Bats	706	Faeces	Ashanti, Bono	706
Total				**1094**

The archived viral RNA extracts were previously obtained by the Qiagen Viral RNA Mini Kit protocol according to the manufacturer’s instructions (Qiagen GmbH, Hilden, Germany) for the livestock samples and the MagNA Pure 96 Viral Nucleic Acid Small Volume Kit (Roche Holding AG, Basel, Switzerland) for the bats, grasscutters, and rabbits.

**Fig. 2:**
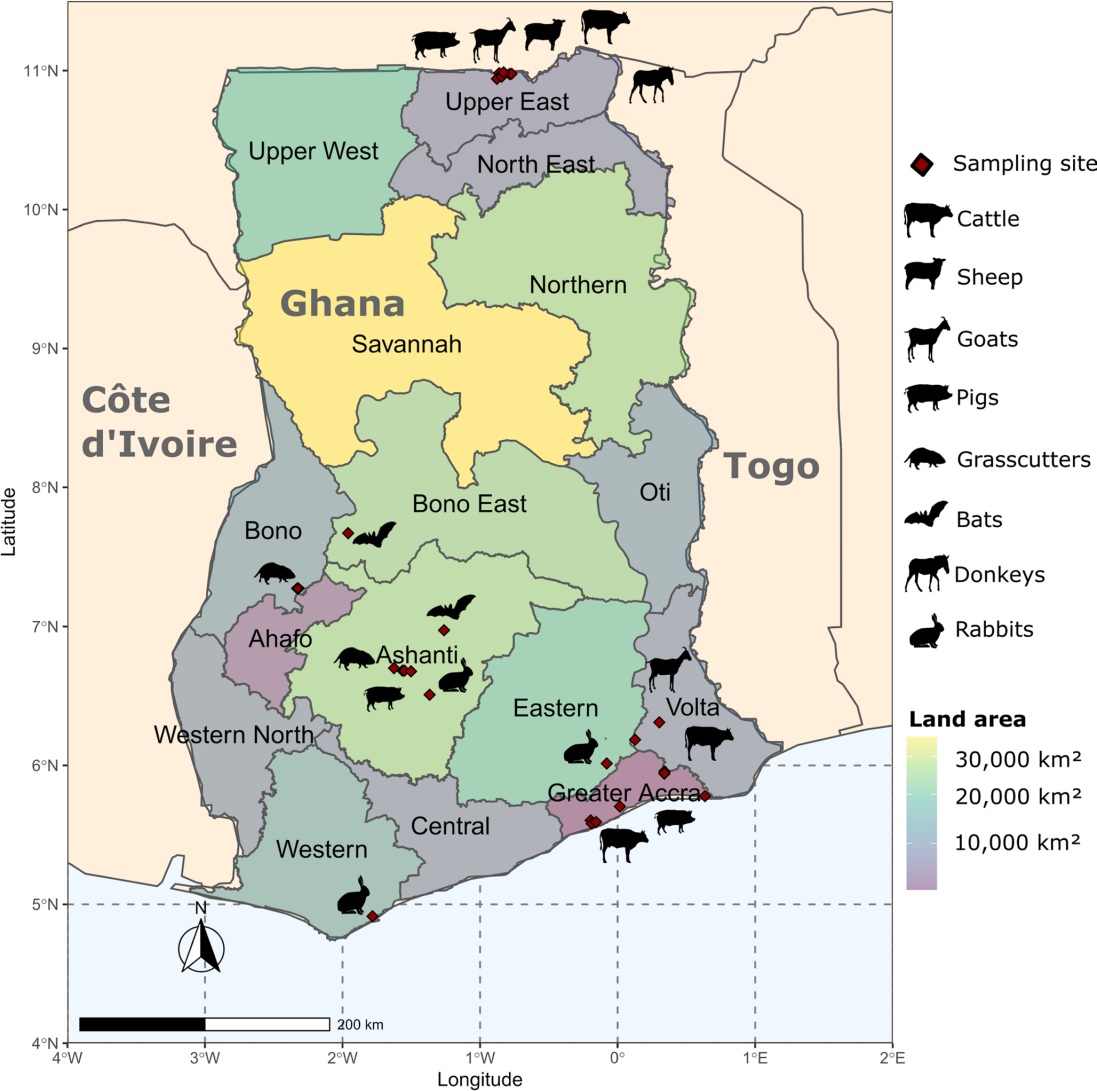
Map of Ghana showing sampling locations of archive samples used in the study. The map was generated using the R statistical package version 4.2.1. Sampling locations are indicated by diamonds, and the types of animals sampled in particular regions are also shown

The distribution of sampling locations is shown in Fig. 2. Bat and livestock samples were tested in pools of 6 and 7, respectively. Pools were made by sample and species type, comprising only faecal extracts. Although sample pooling is effective in saving resources when testing large numbers of samples, the dilution of RNA has the potential to reduce the sensitivity of detection in a low-prevalence setting. Positive pools were resolved for further screening and sequencing. Grasscutter and rabbit samples were tested individually. Confirmation of positive outcomes was done by Sanger sequencing (Microsynth Seqlab GmbH, Göttingen, Germany) and BLAST analysis.

### Analysis of sequences

**Fig. 3:**
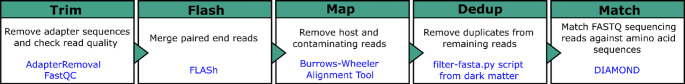
Schematic representation of the steps involved in the bioinformatics analysis of sequencing reads. The software used at each step is shown in blue text, and the description of the step is shown in black text. Software versions were v2.3.2 for AdapterRemoval, v0.11.9. for FastQC, v1.2.11. for FLASh, v0.7.17 for Borrows-Wheeler Alignment Tool, and v2.0 for DIAMOND

Initial assessment of Sanger sequences was performed by neighbor-joining phylogenetic analysis in Geneious using a Tamura-Nei genetic distance model and 500 bootstrap replicates together with similar sequences obtained by BLAST analysis. A subset of samples with clear, distinct bands were selected for complete genome sequencing and characterisation. This was done by a high-throughput sequencing (HTS) approach using the KAPA RNA Hyper Prep Kit (Roche Molecular Diagnostics, Basel, Switzerland) for library preparation. The 150-cycle NextSeq reagent v2 cartridge (Illumina, San Diego, California, USA), was used according to manufacturer’s instructions. Sequencing reads were analysed by an established bioinformatics pipeline available at the Institute of Virology, Charité. The first step after run completion was read trimming and quality control, followed by steps to remove unwanted and duplicate reads. The main analytical part of the process was running the Double Index Alignment of Next-generation sequencing Data (DIAMOND) tool iteratively against multiple curated databases for viruses. The targets included both large and small genome DNA and RNA viruses. The analytical steps and tools used are outlined in Fig. 3. Parameters for use of software were as recommended by the developers for AdapterRemoval (https://adapterremoval.readthedocs.io/en/2.3.x/), FastQC (https://www.bioinformatics.babraham.ac.uk/projects/fastqc/), FLASh (https://ccb.jhu.edu/software/FLASH/), Burrows-Wheeler Alignment Tool (https://bio-bwa.sourceforge.net/bwa.shtml), dark matter (https://github.com/acorg/dark-matter), and DIAMOND (https://github.com/bbuchfink/diamond).

HTS reads were assembled against reference sequences from GenBank and annotated using Geneious. Read mapping for consensus sequence generation was done in stages. The first step was to iteratively map to a closely related reference sequence to obtain partial genomes. Subsequent mappings were done without trimming to the reference, and with multiple iterations until gaps in the consensus were filled. The threshold for consensus base-calling was set to 60% of the total adjusted quality. The genomes were annotated using Geneious to find all open reading frames (ORFs), and using the most closely related sequences on a nucleotide level to each of the genomes as references to assign corresponding ORFs and coding DNA sequences (CDS).

Sequence alignments were made using MAFFT and checked for recombination patterns using Recombination Detection Program version 4.9.7 (RDP4). Whole nucleotide genomes of alphacoronaviruses were assessed by Maximum likelihood phylogenetic analysis using a General Time Reversible model with a gamma distribution and proportion of invariable sites (GTR + G + I) for nucleotide alignments. The PHYML plugin in Geneious [31] was used with 500 bootstrap replicates for this analysis. Complete spike proteins of betacoronaviruses were phylogenetically analysed by Bayesian inference using the MrBayes plugin in Geneious [32]. For protein analysis, nucleotide sequences were translated and aligned with MAFFT and a BLOSUM62 scoring matrix. The Whelan and Goldman (WAG) General Empirical Model was used with a chain length of 1.1 million, a sub-sampling frequency of 200, and 11% burn-in.

Assignment of the detected virus into a subgenus was done according to the demarcation criteria of the International Committee on Taxonomy of Viruses (ICTV) [33]. Briefly, this involved rooted phylogenetic assignment and calculation of pair-wise evolutionary distances for the following conserved domains in the replicase polyprotein pp1ab: ADRP, nsp5 (3CLpro), nsp12 (RdRp), nsp13 (Hel), nsp14 (ExoN), nsp15 (NendoU), and nsp16 (O-MT). This was done using PHYML as previously described above. Viruses with more than 90% amino acid (aa) sequence identity in a distance matrix of the conserved replicase domains were considered to belong to the same species. Viruses that clustered with members of any other genus and share more than 46% sequence identity in the previously mentioned conserved domains were considered to be members of the same genus. Given that the phylogenies for the assignment of a detected virus into a subgenus, coupled with a complete spike phylogeny, provide placement information based on more than two-thirds of the entire genome, a complete genome phylogeny was not further estimated in this case.

## Results

### Detection capability of hemi-nested PCR

**Fig. 4:**
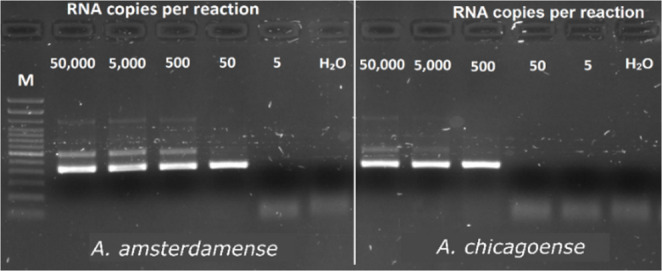
Gel electrophoresis image showing the detection limit of the assay. Values are shown as copies/reaction for *A. amsterdamense* and *A. chicagoense *in vitro transcripts. Each reaction was performed in conjunction with a negative control using water as a template. M depicts the 100 bp molecular weight standard (Invitrogen) for estimation of band sizes. A positive reaction is depicted by a band size of approximately 419 bp

**Fig. 5:**
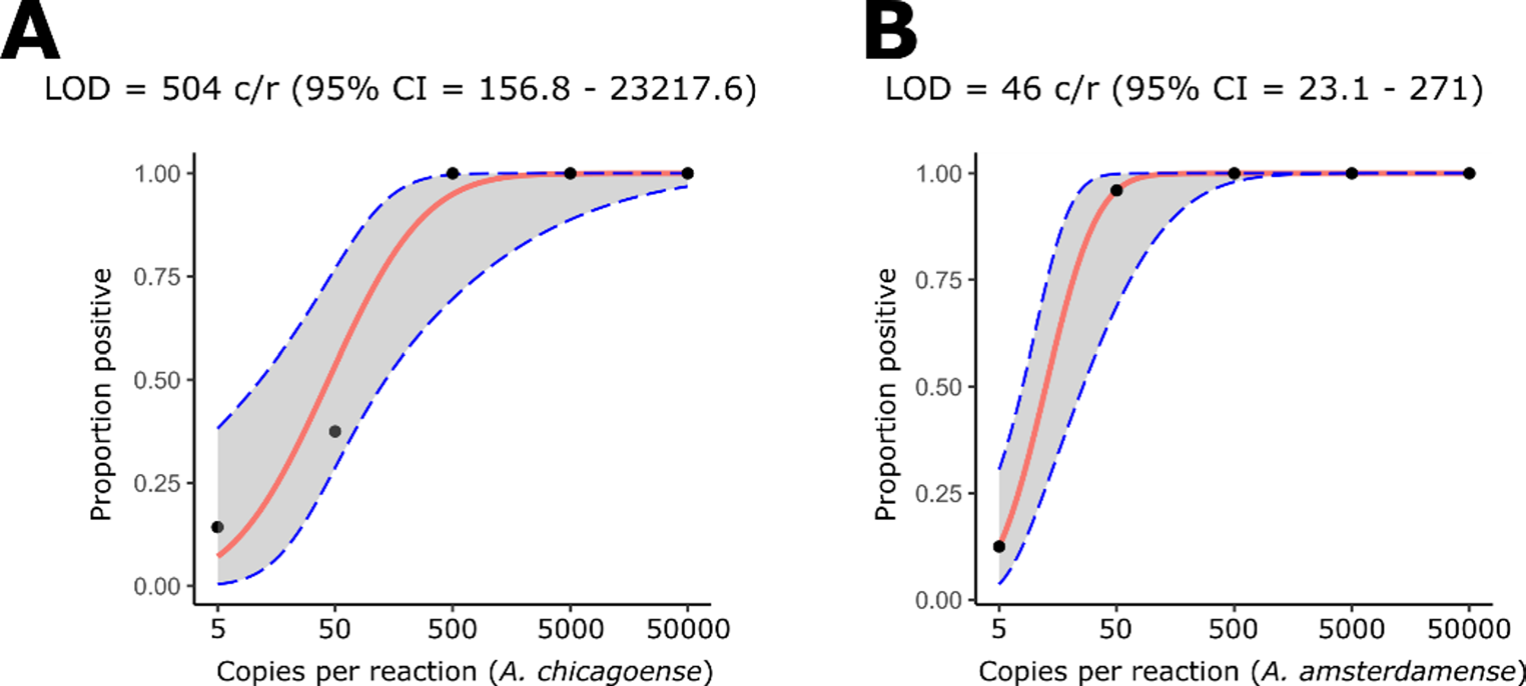
Probit regression analysis of in vitro transcript dilution series. Panel **A** shows the limit of detection for *A. chicagoense*, and panel **B** shows that for *A. amsterdamense*. The red line depicts the probit regression model, and the blue dotted line shows the confidence limits. Black dots show probabilities of positive PCR outcomes at different dilutions of the in vitro transcripts

There was constant detection at 50 copies/reaction of the *A. amsterdamense* IVT and 500 copies/reaction of the *A. chicagoense* IVT (Fig. 4). The assay was found to be able to detect at least 46 copies/reaction of the *A. amsterdamense* and 504 copies/reaction of the *A. chicagoense* IVTs as determined by probit regression analysis of 8 replicates at each dilution (Fig. 5). In silico analysis of the nested primer showed binding to all alphacoronaviruses with a minimum of 3 mismatches away from the 3′ end. The nested primer showed increasing binding capacity across a broader diversity of betacoronaviruses as additional mismatches were allowed. When mismatches away from the 3′ end were permitted, nested primers bound to 2 of 69 sequences with three mismatches, 8 of 69 with four mismatches, and 27 of 69 with five mismatches.

### Outcomes of RT-PCR testing of archived samples

No coronaviruses were detected in any of the domestic and peri-domestic livestock samples tested. Of the 706 bat samples tested, 36 (5.1%) were positive by RT-PCR, of which 22 samples (3.11% of all bats) were confirmed by Sanger sequencing. Not all PCR-positive samples were successfully sequenced, most likely due to low viral loads. Sequences obtained ranged in length from 353 to 398 bp. The positive samples comprised 13 alphacoronaviruses (1.84%) and 9 betacoronaviruses (1.27%) from different bat species (Online Resource 4). Among the alphacoronaviruses, two groups were observed; the first group comprised 11 of the 13 viruses, which were isolated from *Hipposideros* sp. bats. These viruses shared the highest pairwise identity of 96.41% with a virus from Kenya (accession number KY073748). The second group comprised the remaining 2 out of 13 viruses. These viruses were most closely related to *Rousettus* sp. alphacoronaviruses from South Africa, with pairwise sequence identity of 96.1% (accession numbers MZ547451 and MZ547452), and a *Chaerephon* spp. bat coronavirus from Kenya with pairwise identity of 97.34% (accession number KX284952).

The detected betacoronaviruses shared the highest sequence identity with a *Hipposideros* sp. bat coronavirus from Ghana, with pairwise sequence identity of 98.93% (accession number FJ710047).

Eleven of the bat samples tested were part of a collection that previously tested positive with a combination of generic and specific PCR assays [12]. The developed assay in this study detected 7 out of the 11 previous positives. The remaining 15 samples that tested positive in this study were from archive samples unique to this study.

### Complete genome generation 

All sequences obtained in this study have been deposited in GenBank and assigned accession numbers PP182442 - PP182444 for the complete genomes and PP238490 - PP238508 for the partial sequences. Raw sequencing reads were also deposited in the NCBI Sequence Read Archive with BioSample accession numbers SAMN54949924 for sequence PP182444, SAMN54949925 for sequence PP182442, and SAMN54949926 for sequence PP182443. All the raw read data can be found under BioProject number PRJNA1415830. We obtained complete genomes for the previously reported partial genomes of *A. chicagoense*-related Alphacoronavirus FJ710044 (BtCoV/Hip/344/2008/Ghana), henceforth referred to by its accession number PP182442, and Betacoronavirus FJ710043 (BtCoV/Hip/348/2008/Ghana), henceforth referred to as PP182443, from *Hipposideros* sp. bats. A near-complete genome for an additional Alphacoronavirus (BtCoV/Tad/249/2008/Ghana), henceforth referred to as PP182444, was also detected in this study from a *Tadarida major* bat (*Chaerephon* spp.). The parameters of the complete and near-complete genomes generated in the study are shown in Table 2.

**Table 2:** Overview of complete and near-complete genomes generated in the study

Parameter	Sequence name		
	PP182442	PP182443	PP182444
Host species	*Hipposideros sp.*	*Hipposideros sp.*	*Tadarida major*
Number of read pairs (Million)	12.9	11.5	9.1
Reference for mapping	KT253271	HQ166910	OQ792155
Number of reads matched to reference sequence (Thousand)	599,982	7200	2,087
Percentage of genome covered	100%	100%	97.3%
Mean read coverage	2103.5	71.3	5.6
Most closely related sequence (Nucleotide)	KT253271	PP273171	OQ792155
Sequence identity to the most closely related complete sequence	94.19%	80.5%	95.3%

### Genome annotation and characterisation

**Fig. 6:**
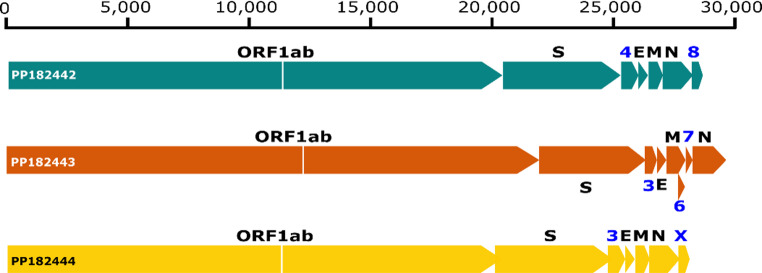
Genome organization of coronaviruses detected in the study. Green represents the PP182442 alphacoronavirus, yellow represents the PP182444 *Chaerephon* spp. alphacoronavirus and orange represents the PP182443 betacoronavirus. ORF1ab refers to the overlapping open reading frames 1a and b. S: spike protein, E: envelope protein, M: Matrix protein, and N: nucleocapsid protein. Blue text represents open reading frames other than 1ab

All three complete genomes exhibited the typical coronavirus genome architecture comprising open reading frame 1ab (ORF1ab), spike, membrane, envelope, and nucleocapsid structural proteins. The PP182442 *A. chicagoense*-related Alphacoronavirus complete genome was 28,757 bp long and included an ORF4 and a terminal 3’ ORF8. The near-complete PP182444 *Chaerephon* spp. alphacoronavirus genome was 27,841 bp long with a 97.3% genome coverage, and included an ORF3 and a terminal 3’ ORFx. The ORFx label functions as a neutral placeholder for the uncharacterised accessory gene with no specific functional name. The complete genome of the PP182443 Betacoronavirus sequenced in this study was 30,123 bp long and included ORFs 3, 6, and 7 (Fig. 6). The differences in accessory ORFs observed between the genomes are typical in the evolution of coronaviruses, reflecting host or lineage-specific adaptations.

Recombination analysis did not show any events for any of the genomes from this study using RDP, GENECONV, Chimera, MaxChi, and 3Seq analysis among the viruses in the alignments used for phylogenetic analysis.

**Fig. 7:**
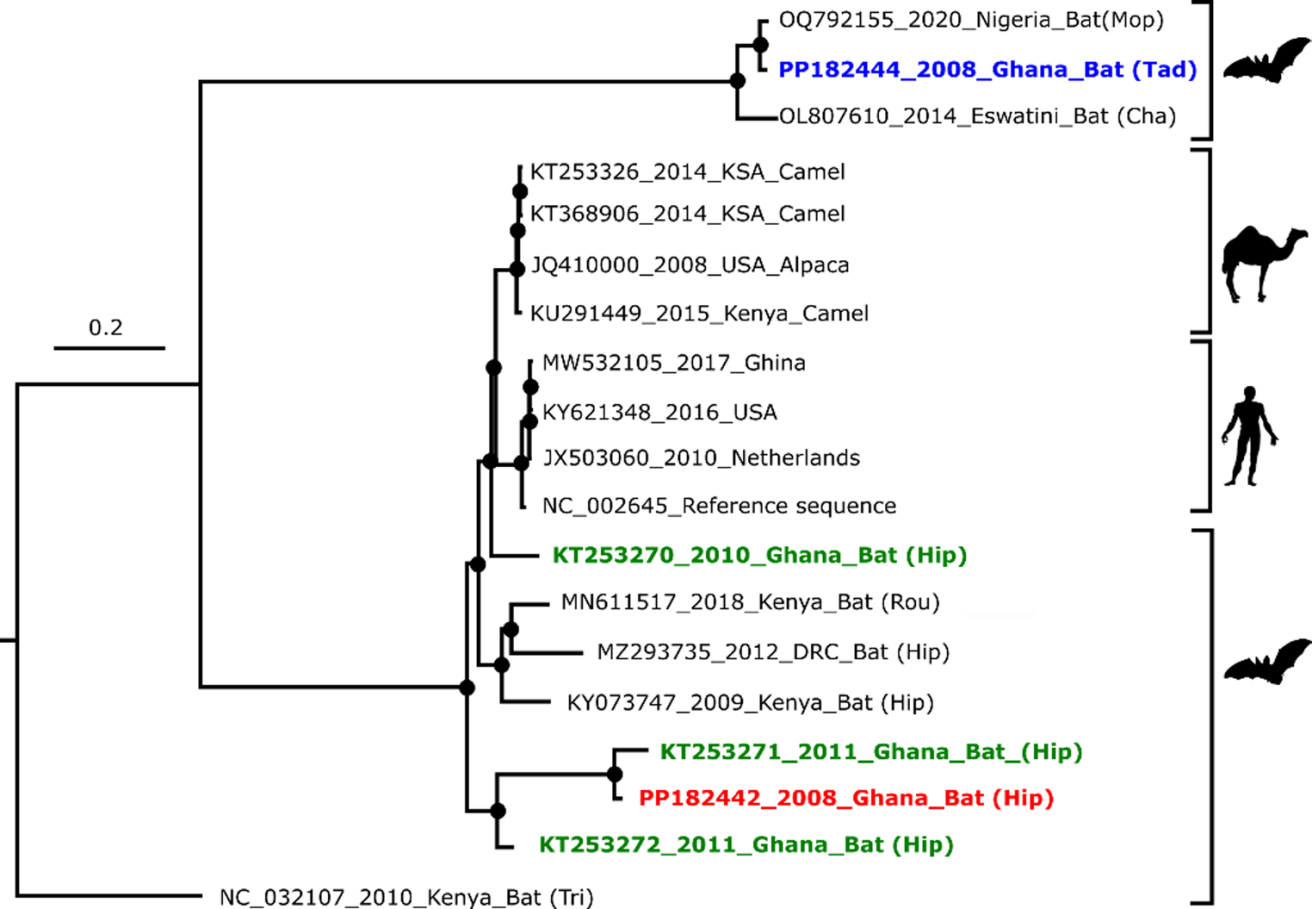
Phylogenetic placement of alphacoronaviruses detected in this study. Complete genome phylogenetic analysis of alphacoronaviruses obtained in this study, together with other coronaviruses obtained from GenBank. The PP182442 alphacoronavirus is shown in red font, and the PP182444 alphacoronavirus is shown in blue. Other *A. chicagoense* - related coronaviruses from Ghana are shown in green. Bootstrap replicates 70% or greater are shown with black dots, and the tree is rooted with an *A. amsterdamense* - related bat coronavirus from Kenya. Sequences are represented by accession numbers, year of isolation, country of origin, and host species. For bats, the first three letters of the Genus names are also included for further differentiation, with the representations being Hip for *Hipposideros* sp., Tad for *Tadarida* sp., Mop for *Mops* sp., Cha for *Chaerephon* sp., and Rou for *Rousettus* sp.

**Fig. 8:**
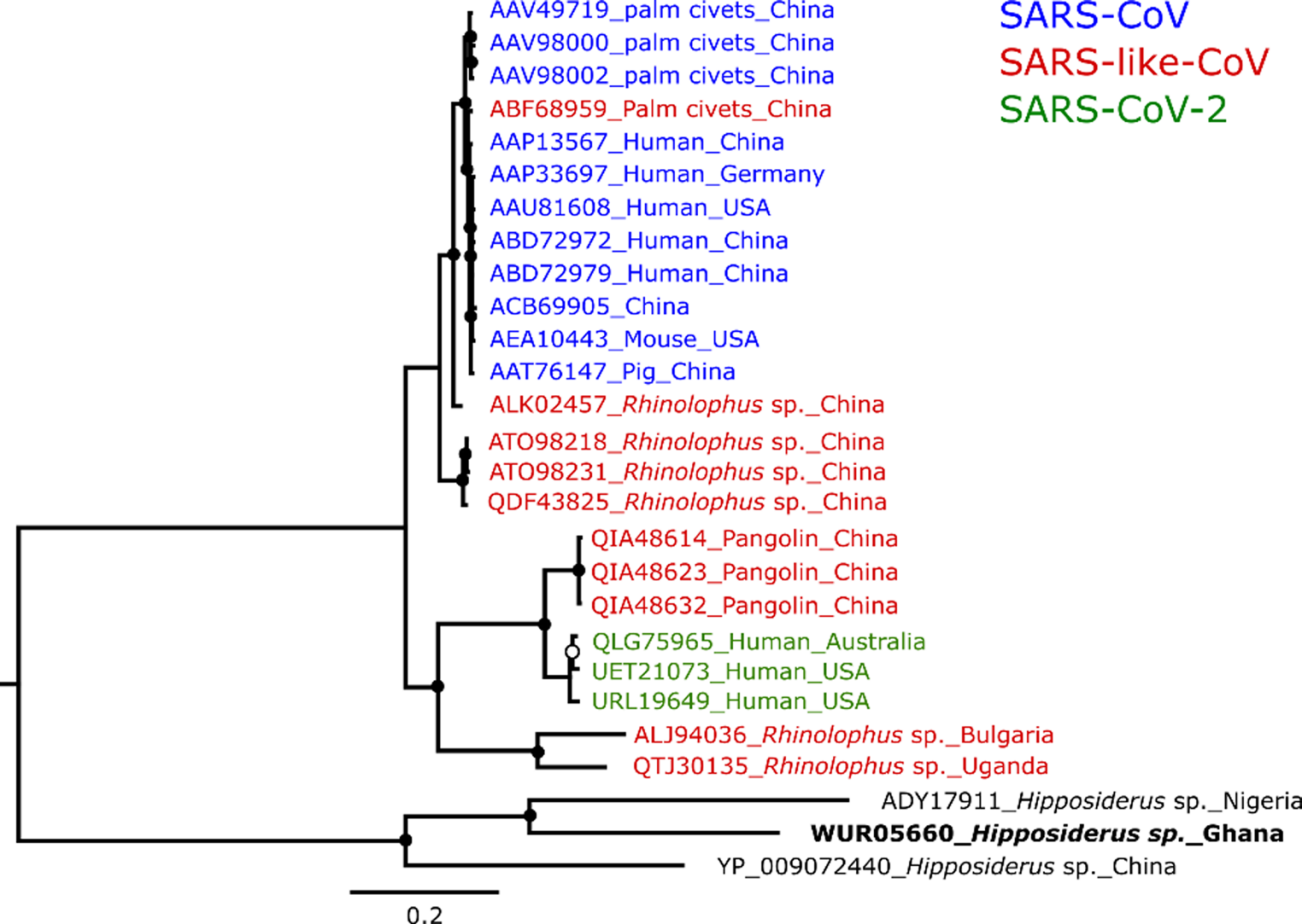
Phylogenetic placement of the betacoronavirus detected in this study. Complete spike amino acid phylogenetic analysis of the PP182443 betacoronavirus obtained in this study is depicted in black and bold font. Blue sequences are SARS coronaviruses, red sequences are SARS-like coronaviruses, and green sequences are SARS-CoV-2 sequences. The white circular node represents a posterior probability value less than 0.95, and black circular nodes represent posterior probabilities greater than 0.95. The tree is rooted with a *Hibecovirus* monophyletic group. Sequences are represented by protein accession numbers, country of origin, and host species

The complete genome of the *A. chicagoense*-related Alphacoronavirus clustered with and was found to be most closely related to another one from Ghana (accession number: KT253271) collected in 2011, with pairwise sequence identity of 94.19%. The *Chaerophon* spp. Alphacoronavirus clustered with and was determined to be most closely related to a *Mops condylurus* alphacoronavirus (accession number: OQ792155) collected in Nigeria, with a pairwise sequence identity of 95.3% (Fig. 7). Phylogenetic analysis of the complete spike aa sequence of the detected betacoronavirus compared to other closely related viruses showed this virus to be most closely related to another *Hipposiderus* sp. betacoronavirus from Nigeria, with pairwise aa sequence identity of 55.81% (accession number ADY17911) as seen in Fig. 8.

**Fig. 9:**
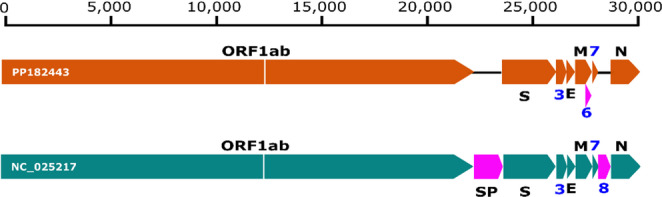
Genome comparison of hibecovirus from this study to an Asian isolate. Orange represents the PP182443 genome obtained in this study, while green represents an Asian hibecovirus reference genome (NC_025217). Purple represents ORFs missing from the counterpart genome. ORF1ab refers to the overlapping open reading frames 1a and b. S: spike protein, SP: putative surface protein, E: envelope protein, M: Matrix protein, and N: nucleocapsid protein. Blue text represents open reading frames other than 1ab. Text within the blocks is the specific virus isolate names

The detected PP182443 betacoronavirus clustered with another coronavirus belonging to the subgenus *Hibecovirus*, which has only one species, *Betacoronavirus hipposideri* (*Bat Hp-betacoronavirus Zhejiang2013*, accession number: NC_025217). The clustering was seen in different phylogenies of six subsections of the replicase polyprotein pp1ab (see Online Resource 5). PP182443 and *B. hipposideri* shared the highest aa sequence identities of 43.7% in the nsp5, 53.4% in the nsp12, 90% in the nsp13, 88.7% in the nsp14, 81% in the nsp15, and 80.9% in the nsp16. The complete genome presented here represents one of the few of African origin, and represents a significant contribution to our knowledge of the diversity of African betacoronaviruses. PP182443 shows differences to *B. hipposideri* (accession number: NC_025217) and another available complete genome from Asia isolated from a *Hipposideros pratti* bat (accession number: OQ175261). Our complete genome shares 80.5% sequence identity with one from Kenya [34] and 63.9% sequence identity with the Asian ones, which are 97.6% similar to each other on the nucleotide level. The larger differences from the Asian isolates are mainly due to the fact that the sequence from this study lacks the putative surface protein CDS preceding the spike, and also appears to lack the ORF8, both of which are found in the Asian sequences. The Asian sequences in turn appear to lack the putative ORF6 found in our PP182443 sequence (Fig. 9). The observed absence of these proteins in PP182443 in comparison to the Asian sequences suggests significant divergence between these geographically separate viruses.

## Discussion

Regular surveillance and characterisation of coronaviruses are essential for identifying those with zoonotic potential and for advancing our understanding of their evolutionary dynamics. While several assays are designed for broad coronavirus detection [35–37], the more targeted assay established in this study enhances the likelihood of detecting specific members of the family, such as alphacoronaviruses that occur in humans, livestock, and wildlife [38]. Owing to the conserved nature of the selected genomic region (nsp12), the assay also retains the capacity to detect emerging variants. The observed detection capability for betacoronaviruses can further be attributed to the conserved nature of the nsp12 target, even though the nested primer was designed primarily for alphacoronaviruses. However, detection across a broader diversity of betacoronaviruses may be limited, as indicated by in silico analyses. This broad detection capability which underscores the high conservation of the nsp12 region is consistent with other studies that have established genus-level PCR assays [35, 39]. The assay described here provides a sensitive platform for identifying reservoir and intermediate hosts of coronaviruses, such as *A. amsterdamense*, for which no intermediate host has yet been identified.

The absence of detections among livestock screened during assay validation is unsurprising, as this study was not designed to determine baseline prevalence of coronaviruses in domestic animals. The sample size in this study was insufficient to provide robust estimates of baseline infection rates in clinically healthy livestock, and the testing performed on pooled samples further reduces the probability of detecting low-level circulating viruses. Furthermore, coronaviruses are rarely detected outside of outbreak contexts [40, 41]. Concentrations of pathogenic alphacoronaviruses in livestock with clinical disease have been shown to be high in prior studies, ranging from approximately 10^7^ copies/mL for *A. chicagoense* – related viruses in camel respiratory swabs [17] to 10^6^ copies per gram of faeces for *A. porci* in swine [42]. Given the performance characteristics of the assay, it is reasonable to conclude that it can detect alphacoronaviruses at these levels, as well as those circulating in reservoir hosts where lower viral loads are typically expected [43]. Faecal samples were prioritized for assay validation due to their non-invasive collection from animals; which enables easier comparative population-level estimates across livestock and wildlife, their prolonged virus shedding duration compared to respiratory samples [44, 45], and established use as the primary diagnostic matrix for asymptomatic animal coronavirus surveillance [46–48].

In bats, alphacoronaviruses that are primarily found in *Chaerephon* spp. are widely distributed across Africa, with detections from Nigeria in the west, the Democratic Republic of Congo in the central part, and Eswatini in the southern part of the African continent [49, 50]. *Chaerephon* bats are synanthropic, often roosting in close proximity to humans (e.g., building roofs) [51]. This behavior increases the likelihood of interactions with humans and livestock, thereby elevating the risk of spillover events. Despite this proximity and spillover risk, phylogenetic clades of bat coronaviruses remain separated from those of humans and domestic animals [52], suggesting either spillover is not frequent enough or the existence of barriers preventing sustained cross‑species transmission. The host-driven separation of clades observed in both the alpha- and betacoronavirus phylogenetic trees in this study, where bat viruses cluster distinctly from those infecting other hosts, is consistent with the substantial ecological and evolutionary diversity documented in African bat populations.

Although surveillance studies have historically prioritized bats [53], expanding such efforts to livestock is essential for monitoring potential cross-species transmission at the human-animal interface. The high sequence identity between the PP182444 *Chaerephon* bat alphacoronavirus reported here and its closest relative from Nigeria aligns with findings from Kenya, which showed African *Chaerephon* alphacoronaviruses to be more closely related to each other than to strains outside the continent [54]. This pattern suggests interconnected bat populations and shared viral ancestry within Africa.

The assay also demonstrated the ability to detect co-circulating betacoronaviruses, including a putative member of the subgenus *Hibecovirus*. The hibecoviruses represent a substantial component of West African bat coronaviruses [55], predominantly detected in *Hipposideros* spp. and, to a lesser extent, *Rhinolophus* spp. bats [56]. Phylogenetic analyses position hibecoviruses as a sister clade to the sarbecoviruses, which include SARS-CoV and SARS-CoV-2, making them the closest known relatives [53]. The betacoronavirus sequenced in this study clustered with the only described member of the subgenus, and aside from nsp5, shared > 46% amino acid identity across conserved replicase domains used for genus and species demarcation [1]. These findings support its classification within the genus *Hibecovirus.*

There was a geographically structured difference between the hibecovirus isolated in this study and its Asian counterparts. Certain viral lineages, particularly in African bats, show regional clustering due to limited mixing across the continent. Many bat species display strong roost fidelity, repeatedly using the same sites with little colony switching, which restricts virus transmission and gene flow between distant populations [55]. Short foraging ranges and limited long-distance movement further reduce opportunities for virus spread beyond local areas. In addition, long-term co-divergence between bats and their coronaviruses promotes lineage-specific adaptations, limiting cross-population jumps and preserving geographic patterns in bat-borne viral lineages [55, 57].

In cohabiting bat species, evidence of host switching raises the potential for recombination, which can increase viral diversity [58]. Although no direct *Hibecovirus* spillover to humans has been documented in Africa, their close relationship to sarbecoviruses, a lineage that includes SARS-CoV-2, underscores the importance of continued monitoring. Despite being the third most frequently detected betacoronavirus lineage in bats, following nobecoviruses and sarbecoviruses [56], few complete *Hibecovirus* genomes are currently available apart from the reference sequence *Bat Hp-betacoronavirus Zhejiang2013*. This scarcity reflects limited sequencing efforts relative to other betacoronavirus groups [58], highlighting the urgent need for expanded genomic surveillance.

Enhanced, targeted surveillance aimed at recovering complete genomes from natural reservoirs and potential intermediate hosts will be critical for resolving the evolutionary history of underrepresented coronaviruses such as hibecoviruses. The geographic variation observed in this study further reinforces the necessity of comprehensive monitoring across Africa to better capture the diversity of this group within the *Coronaviridae* family.

This study has some limitations, the first being the use of archived RNA samples. The archived sample collections used in this study were opportunistic and lacked sufficient contextual data to allow precise estimates of incidence or prevalence. In addition, the sample pooling reduces detection sensitivity and may have hindered full-genome recovery for some viruses. Although a near-complete genome was obtained for the PP182444 alphacoronavirus sequence, overall coverage was low and may have limited the detection of low-frequency mutations.

## Conclusion

Screening assays are critical for monitoring coronaviruses circulating at the human-animal interface and for elucidating their host range. The assay evaluated in this study exhibited strong detection performance and proved highly effective for coronavirus surveillance. The assay facilitated the identification and recovery of complete and near-complete genome sequences from bat alpha- and betacoronaviruses, thereby contributing to a deeper understanding of the diversity and evolutionary dynamics of African coronaviruses.

## Supplementary information

Below is the link to the electronic supplementary material.

Supplementary Material 1

Supplementary Material 2

Supplementary Material 3

Supplementary Material 4

Supplementary Material 5

## Data Availability

All data generated or analysed during this study are included in this manuscript and in the publicly available National Center for Biotechnology Information (NCBI) database, with accession numbers provided in the manuscript.
