# Supplementary material for: Detection and complete genome characterisation of bat coronaviruses from Ghana
Source: Arch Virol. 2026 May 21;171(6):188. doi: 10.1007/s00705-026-06628-y (PMC13194324; doi:10.1007/s00705-026-06628-y)
Supplement: Supplementary file 4 — Supplementary Material 4 [file 705_2026_6628_MOESM4_ESM.pdf]

Archives in Virology

Detection and Complete Genome Characterization of Bat Coronaviruses from Ghana

Philip El-Duah[1✉], Richmond Yeboah[2], Julia Melchert[1], William Tasiame[3], Emmanuella Nyarko-Afriyie[2], Augustina Sylverken[4], Michael Owusu[5], Yaw Adu-Sarkodie[6], Christian Drosten[1], Victor Max Corman[1✉]

1. Institute of Virology, Charité-Universitätsmedizin Berlin, Corporate member of Free University, Humboldt-University and Berlin Institute of Health, Berlin, Germany
2. Kumasi Centre for Collaborative Research in Tropical Medicine, Kwame Nkrumah University of Science and Technology, Kumasi, Ghana
3. School of Veterinary Medicine, Kwame Nkrumah University of Science and Technology, Kumasi, Ghana
4. Department of Theoretical and Applied Biology, Kwame Nkrumah University of Science and Technology, Kumasi, Ghana
5. Department of Medical Diagnostics, Kwame Nkrumah University of Science and Technology, Kumasi, Ghana
6. Department of Clinical Microbiology, Kwame Nkrumah University of Science and Technology, Kumasi, Ghana

Corresponding author: philip.el-duah@charite.de, victor.corman@charite.de

Table 1. Distribution of detected coronaviruses by bat species

		Sequence-confirmed detections		
Species	Number of samples	AlphaCoV	BetaCoV	Combined
Coleura afra	71			
Eidolon helvum	220			
Epomophorus gambianus	6			
Epomops buettikoferi	1			
Epomops franqueti	1			
Glauconycteris beatrix	1			
Hipposideros abae	81	1	1	2
Hipposideros cf caffer	20			
Hipposideros ruber	124	7	2	9
Hipposideros spec	80	3	6	9
Hypsignathus monstrosus	1			
Micropteropus pusillus	13			
Micropteropus/Nanonycteris	2			
Nanonycteris veldkampi	23			
Nycteris hispida	1			
Nycteris spec	3			
Pipistrellus deserti	1			
Pipistrellus nanulus	2			
Pipistrellus nanus	8			
Pipistrellus spec	6			
Rhinolophus landeri	1			
Rousettus aegyptiacus	32	1		1
Tadarida major	7	1		1
Tadarida spec	1			
Total	706	13	9	22
