# Supplementary material for: Detection and complete genome characterisation of bat coronaviruses from Ghana
Source: Arch Virol. 2026 May 21;171(6):188. doi: 10.1007/s00705-026-06628-y (PMC13194324; doi:10.1007/s00705-026-06628-y)
Supplement: Supplementary file 5 — Supplementary Material 5 [file 705_2026_6628_MOESM5_ESM.pdf]

Archives in Virology

Detection and Complete Genome Characterization of Bat Coronaviruses from Ghana

Philip El-Duah[1]✉, Richmond Yeboah[2], Julia Melchert[1], William Tasiame[3], Emmanuella Nyarko-Afriyie[2], Augustina Sylverken[4], Michael Owusu[5], Yaw Adu-Sarkodie[6], Christian Drosten[1], Victor Max Corman[1]✉

1. Institute of Virology, Charité-Universitätsmedizin Berlin, Corporate member of Free University, Humboldt-University and Berlin Institute of Health, Berlin, Germany
2. Kumasi Centre for Collaborative Research in Tropical Medicine, Kwame Nkrumah University of Science and Technology, Kumasi, Ghana
3. School of Veterinary Medicine, Kwame Nkrumah University of Science and Technology, Kumasi, Ghana
4. Department of Theoretical and Applied Biology, Kwame Nkrumah University of Science and Technology, Kumasi, Ghana
5. Department of Medical Diagnostics, Kwame Nkrumah University of Science and Technology, Kumasi, Ghana
6. Department of Clinical Microbiology, Kwame Nkrumah University of Science and Technology, Kumasi, Ghana

Corresponding author: philip.el-duah@charite.de, victor.corman@charite.de

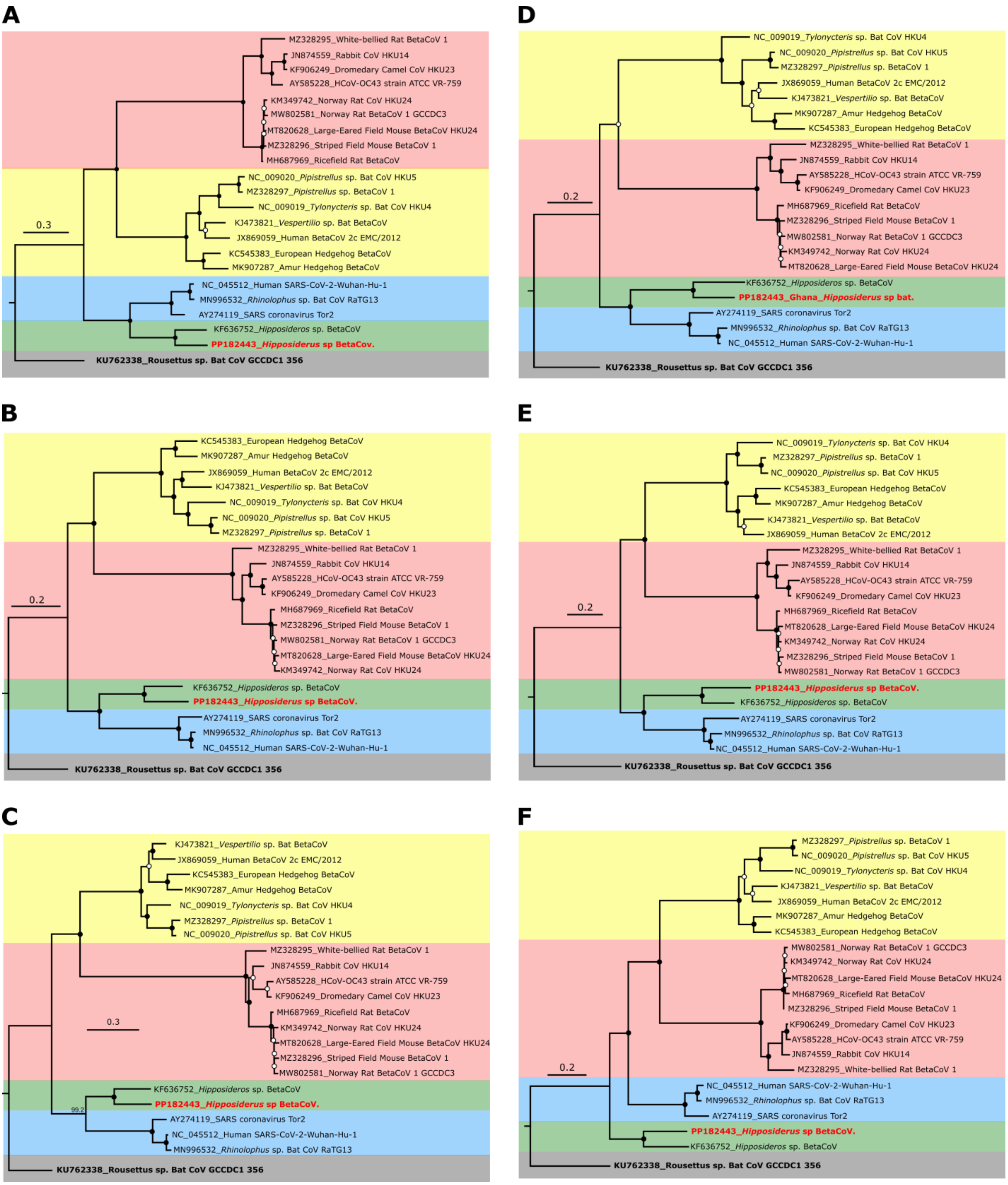

Figure S1. Phylogenetic placement of detected betacoronavirus in different parts of the ORF1ab.

Panels A to F represent nsp 5, 12, 13, 14, 15, and 16, respectively. Color palette represents selected members of the subgenera: Yellow for *Merbecovirus*, pink for *Embecovirus*, blue for *Sarbecovirus*, gray for *Nobecovirus* which was used to root the tree, and green for *Hibecovirus*. The sequence obtained in this study is indicated with red font. Trees were generated by Maximum likelihood estimation with PHYML. Black nodes represent bootstrap replicate values of 70% or greater, and white nodes represent those with lower values. Sequences are represented by Accession numbers and virus descriptions.
